# *In vitro* and *in vivo* study: Ethanolic extract leaves of *Azadirachta indica* Juss. variant of Indonesia and Philippines suppresses tumor growth of hepatocellular carcinoma by inhibiting IL-6/STAT3 signaling

**DOI:** 10.12688/f1000research.109557.1

**Published:** 2022-04-29

**Authors:** Ricadonna Raissa, Wibi Riawan, Anna Safitri, Masruri Masruri, Ma Asuncion Guiang Beltran, Aulanniam Aulanniam

**Affiliations:** 1 Doctoral Program of Chemistry, Faculty of Mathematics and Natural Science, Universitas Brawijaya, Malang, East Java, Indonesia; 2 Department of Biochemistry, Faculty of Medicine, Universitas Brawijaya, Malang, East Java, Indonesia; 3 Department of Chemistry, Faculty of Mathematics and Natural Science, Universitas Brawijaya, Malang, East Java, Indonesia; 4 Research Center for Smart Molecules of Natural Genetic Resources (SMONAGENES), Universitas Brawijaya, Malang, East Java, Indonesia; 5 College of Veterinary Medicine, Tarlac Agricultural University, Camiling, Tarlac, Philippines; 6 Department of Biochemistry, Faculty of Veterinary Medicine, Universitas Brawijaya, Malang, East Java, Indonesia

**Keywords:** Azadirachta indica, cancer immunotherapy, interleukin - 6 (IL - 6), signal transducer activator of transcription 3 (STAT3), vimentin, hepatocellular carcinoma

## Abstract

**Background:** *Azadirachta indica* Juss. has been shown to suppress cancer progression through a variety of mechanisms. In order to treat cancer progression, cancer immunotherapy is used to stimulate the immune system where immunosuppression is present in tumor microenvironments. Many cancer cells produce a lot of interleukin-6 (IL-6) and signal transducer activator of transcription 3 (STAT3). STAT3 plays a key role in suppressing the expression of critical immune activation regulators. IL‐6‐mediated STAT3 activation is common in the tumor microenvironment. Inhibiting the IL-6/STAT3 signaling pathway has become a therapeutic option for cancer progression. As vimentin is also expressed in hepatic stellate cells boosting cancer survival. We focused on the precise effect of extract from leaves of *Azadirachta indica* Juss, on inhibiting the IL-6/STAT3 signaling cascade on hepatocellular carcinoma by *in vitro* and *in vivo* study.

**Methods:** In the *in vitro* study, the effect of *Azadirachta indica* Juss. variant Indonesia and Philippines against the expression of IL-6 and STAT3 was examined in liver cancer cell line. In the *in vivo* study, 24 male rats ( *Rattus norvegicus*) strain Wistar were induced by diethylnitrosamine and carbon tetrachloride (CCl _4_). Based on the therapy given, the groups were divided into negative control, positive control, Indonesia extract, and Philippine extract. Expression of IL-6, STAT3, and vimentin were tested using immunohistochemistry staining. The data were analyzed using analysis of variance, which was then followed by the Tukey test.

**Results:** Statistically significant difference in IL-6 and STAT3 was observed between the treatment groups and positive control group by *in vitro* study and *in vivo* study. Generally, there is no significant difference between treatment using Indonesian and Philippine leaves.

**Conclusion**: Both therapy doses of *Azadirachta indica* variant in Indonesia and Philippines were able to reduce IL-6, STAT3 and vimentin expression of hepatocellular carcinoma cell by *in vitro* and *in vivo* experiment.

## Introduction

Hepatocellular carcinoma (HCC), primary liver cancer originating from hepatocytes, is the fourth most common tumor worldwide. ^1^^,^^2^ From the report of statistics from the Global Burden of Cancer Study, approximately 906,000 new cases and 830,000 deaths of HCC cases were recorded in 2020. ^3^ According to annual projections, more than one million people will die from liver cancer by 2030, according to the World Health Organization. ^4^

HCCs are complex ecosystems that include non-tumor cells, primarily immune-related cells, as well as tumor cells. ^4^^,^^5^ Non-immune and immune cells, cytokines such interleukin-6 (IL-6) and signal transducer and activator of transcription 3 (STAT3) play central roles in inflammation cancer. ^6^^,^^7^ IL-6 belongs to the cytokine family that signals via the Janus kinase-signal transducer and activator of transcription pathway. ^8^^–^^10^ IL-6 has a predominant role in the tumor microenvironment which is found at high concentrations in cancer. ^11^

IL-6 is produced by an HCC cell which functions as a growth factor. ^12^ In addition, it is also reported that hepar cancer produces IL-6, so an increase in IL-6 is a sign of excessive cell growth. ^13^ HCC is caused by abnormal IL-6 signaling in liver progenitor cells with activated STAT3. ^14^ The binding of IL-6 to the IL-6 receptor activates STAT3, a key oncogenic transcription factor. Due to STAT3 critical function in cell signaling, the protein STAT3 has become a popular target in tumor growth. ^15^ Survival of cancer cells can be maintained by the complex IL-6 and STAT3 pathways. ^16^

A study by Iwahasi ^17^ suggested that HCC tumor malignancy, through the IL-6/STAT3 pathway, is affected by the activation of hepatic stellate cells. These hepatic cells boosted cancer cell survival, and migratory ability. Vimentin, in particular, was substantially expressed in activated hepatocyte stellate cells (HSCs). ^18^^,^^19^ As a result, inhibiting HSC cell growth and inhibiting the IL-6/STAT3 pathway could be a promising technique for inhibiting cancer cell progression. ^13^^,^^20^^,^^21^

Cancer treatments that target the IL-6/STAT3 pathway contribute a therapeutic benefit for inhibiting tumor cell growth. ^22^ A study by Kao *et al.* ^23^ has shown that hepatic function, tumor development, and HCC patient survival were all impacted by IL-6 via the STAT3 signal pathway.

Cancer chemotherapy by natural agents is a promising therapy towards lowering cancer progression. ^24^^,^^25^ Overcoming the immunosuppressive condition in tumor microenvironments is significant to improve the efficacy of cancer immunotherapy. ^26^^,^^27^ Immune cells, non-immune cells and tissues, communicate through interleukins and related cytokines. ^28^ Interleukins have a crucial role in the genesis, progression, and management of cancer. Interleukins can create an environment that promotes cancer growth while also being necessary for a successful tumor-directed immune response. ^28^^,^^29^

Over the last several decades, an increasing number of plant-derived products, including *Azadirachta indica* Juss., have been explored, as they are cheap and easy to grow in tropical countries, such as Indonesia and the Philippines. ^30^ We found *Azadirachta indica* Juss. content in the leaf was more in Indonesia and Philippine during September–December, which is the component of the plant with the desired properties for cancer treatment. *Azadirachta indica* Juss. has been reported to promote many biological activities including anticancer. A study by Raissa ^31^ suggested that *Azadirachta indica* Juss. variant Indonesia has more flavonoid content, and *Azadirachta indica* Juss. variant Philippines has more terpenoids content than flavonoid. However, the properties anticancer effect of *Azadirachta indica* Juss. from different region planting suppresses cancer progression by immunotherapy pathway of HCC has not yet been identified. This *in vitro* study uses HepG2 cells. Then, *In vivo* study uses a two weeks old male rat *(Rattus norvegicus)* as hepatocellular carcinoma animal model. Rat prefered for this experimental animal model due to their anatomical and physiological, and genetic similarity to humans. ^32^ In this *in vivo* study prefers male than female rats. Female rats may bring variability results because of the changing hormonal state during the oestrous cycle. ^33^ These animal model use two chemical step, once induction with diethylnitrosamine in neonatal age as agent genotoxic and CCl _4_ as promoter to improve the carcinogenesis in rats. ^34^^–^^36^ This study aimed to evaluate the anticancer properties of ethanolic extract leaves of *Azadirachta indica* Juss. variant of Indonesia and Philippines by inhibiting IL-6/STAT3 signaling.

## Methods

### *Azadirachta indica* Juss. extraction

*Azadirachta indica* leaves were collected from Madura, Indonesia and Camiling, Philippines, taxonomically identified by Laboratory of Taxonomy and Plant Development Structure, Brawijaya University with a certificate number 0238/UN10.F09.42/03/2018 and 0250/UN10.F09.42/03/2019. Air-dried powdered leaves (100 g) were extracted by cold maceration in 80% ethanol (300 ml) and filtered. The supernatant was concentrated with an under pressure rotary evaporator (IKA ^®^ HB 10 digital, Germany) at a temperature not exceeding 55°C. The extracts were stored in an air-tight box.

### Cell lines and culture conditions

HepG2 cell lines were obtained from American Type Culture Collection (ATCC, Cat# HB-8065, RRID:CVCL_0027). The HepG2 cells were grown in Dulbecco's modified eagle medium-high glucose medium (Gibco ^®^, Cat#11965092) supplemented with 10% Fetal Bovine Serum qualified, US origin (Gibco ^®^, Cat#26140087), along with 1% Penicillin-Streptomycin (5,000 U/mL) (Gibco ^®^, Cat#15070063). These cells were maintained at 37°C in a humidified incubator under 95% air atmosphere plus 5% CO _2_ (Heracell™ 150i, Thermo Scientific, Cat#50116048).

### Immunocytochemistry

HepG2 cells with a density of 75 × 10 ^3^ cells/well were grown on coverslips in 24-well plates until 80% confluent. Concentration of 170.105 g/ml ethanolic extract leaves of *Azadirachta indica* Juss. variant of Indonesia and 170.415 g/ml of ethanolic extract leaves of *Azadirachta indica* of variant Philippines were added to the cells in the media. After twenty-four hours post-exposure to both extracts, the media was removed and the plates containing the cells were aspirated with phosphate buffered saline (PBS) (Sigma-aldrich ^®^, Cat#P4417). Cells were fixed with methanol for 10 minutes at 40°C and then washed again with PBS. Cells were added with protein block (Novocastra™, Leica, Cat#RE7102) and washed with PBS. Cells were added primary antibodies IL-6 (Santa Cruz Biotechnology, Cat# sc-57315, RRID:AB_2127596) and STAT3 (Santa Cruz Biotechnology, Cat# sc-8019, RRID:AB_628293) for one hour and washed again with PBS. After incubation with primary antibody, cells were added with Histofine ^®^ (Nichirei Biosciences Cat# 424144, RRID:AB_2868561) for one hour and washed again with PBS. After that, 3,3′-Diaminobenzidine (DAB) solution (Novolink™, Leica, Cat# RE7230-K) was added, and incubated for ten minutes. The DAB solution was washed with aquadest. Furthermore, the Mayer solution was added into the well and then incubated for 10 minutes and then discarded and washed with aquadest. In a light microscope, expressing IL-6 and STAT3 will give a brown color, while those that do not express IL-6 and STAT3 will give a purple/blue color. Immunohistochemistry staining was valued by counting the express protein (brown color) at 20 times field of view at every sample then the data is averaged and processed statistically.

### Measurement of IL-6 levels by enzyme-linked immunosorbent assay (ELISA)

When the cells were 70% confluent, the cells were harvested and treated with ethanolic extract leaves of *Azadirachta indica* of variant Indonesia and Philippines for 24 hours. The medium was transferred to a centrifuge tube. The IL-6 levels were checked using the enzyme-linked immunosorbent assay (ELISA) technique with IL-6 ELISA kit (Elabscience ^®^, Cat#E-EL-H6156) according to each manufacturer’s instructions.

### *In vivo* study design, sample calculation, and hepatocellular carcinoma animal model

The second part of this study is an *in vivo* laboratory study with 24 male rats ( *Rattus norvegicus)* strain Wistar that induced with two step chemical hepatocellular carcinoma induction, using diethylnitrosamine (DEN) and carbon tetrachloride (CCl _4_). This research was conducted from January 2019 to December 2020 at the animal experimental laboratory in Institut Biosains, Universitas Brawijaya, Malang. The experimental protocols were approved by the Ethics Committee of the Institut Biosains, Universitas Brawijaya, Malang with protocol number 1138-KEP-38.

The experimental animals used in this study were male white rats aged two weeks old, with bodyweight (BW) ranging from 20-30 grams. During the experiment and the analysis, no rats were excluded due to illness or any other reasons. All samples were randomly allocated in each group. All samples (n = 24) were divided into four groups consisting of six rats each. The groups were divided into negative control, positive control, ethanolic extract leaves of *Azadirachta indica* Juss. variant of Indonesia treatment, and ethanolic extract leaves of *Azadirachta indica* Juss. variant of Philippines.

Each rat was numbered 1-24 and each cage was marked (A-D) to indicate a different group. After that, randomization was carried out to group each rat by one researcher, and other researchers were blinded until the end of the experiment, so that there was no bias. The sample size was determined by Federer’s formula. A total of 24 rats were separated into four groups, each with six rats.

$$\text{Federer}’s\;\text{formula}:\left(T-1\right)\;\left(N-1\right)>15$$

T = Number of groups

N = Number of rats

$$\left(T-1\right)\;\left(N-1\right)>15$$

Six-one month’s pregnant rats ( *Rattus norvegicus*) (Institut Biosains, Malang, Indonesia) were purchased. After the rat mother gave birth, a total of 24-seven day old male rats were divided from the other seven day old female rats. As these rats were very young, they were kept with their mothers until the end of the weaning period. One mother rat for the six male neonatal rats was kept in one cage. After neonatal rats reach one months old, they are kept in individual cages. All these rats were maintained in Laboratory Animal at Institut Biosains, the University of Brawijaya (Malang, Indonesia). The animals were maintained under a controlled environment with temperature of 23°C ± 1°C, humidity of 55 ± 5% and a 12 h light/dark cycle during the experiment. Rats were fed with yellow corn, pollard, and water *ad libitum.* We provide a pipe for each rat to hide or play to reduce distress.

HCC induction to these experimental animals was done by two step induction. First, the two weeks old male rats were induced by using DEN (Sigma-Aldrich Merck, Cat# N0258) at a dose of 50 mg/kg of body weight for single intraperitoneal injection. Second induction was done when the rats were eight weeks old for intraperitoneal injection of carbon tetrachloride (CCl _4_) (Sigma-Aldrich Merck, Cat# 289116) at a dose of 1 mL/kg of body weight (BW) three times a week for twelve weeks.

The rats in the positive group only received DEN-CCl _4_ induction. In the two curative group, DEN-CCl _4_-induced rats with elevated alpha fetoprotein (AFP) level were given ethanolic extract of *Azadirachta indica* of variant Indonesia (500 mg/kg BW) and ethanolic extract leave of *Azadirachta indica* of variant Philippines (500 mg/kg BW) orally for four weeks every day. After being anesthesia with ketamine (Merck, NMID686C) 75-100 mg/kg BW and xylazine (Xyla ^®^, Interchemie, Cat#IX2) 10 mg/kg BW intraperitoneally, the hepar organ store in neutral buffered formalin solution 10% (Sigma-Aldrich Merck, Cat# HT501128). After being euthanized, paraffin blocks of hepar cancer tissue from each rat were made for immunohistochemistry (IHC) staining.

All efforts were made to ameliorate any suffering of animals.

### Immunohistochemistry

The hepar organs were obtained and fixed in 10% buffered formaldehyde immediately. Then hepar organs were embedded in paraffin. The paraffin-embedded blocks were prepared into five-micrometer serial sections. For histopathological assessment, hepar tissue sections were deparaffinized with xylene. Hepar tissues were rehydrated with distilled water. After that, hepar tissues were added hydrogen peroxide 3% for ten minutes and washed with phosphate buffer saline (PBS). Hepar tissues were added protein block (Novocastra™, Leica, Cat#RE7102) for one hour and washed again with PBS. Then, hepar tissues were added with primary antibody IL-6 (Santa Cruz Biotechnology, Cat# sc-57315, RRID:AB_2127596), STAT3 (Santa Cruz Biotechnology, Cat# sc-8019, RRID:AB_628293), and vimentin (Novus Cat# NBP1-97524, RRID:AB_11190427) for one hour and washed with PBS, then added with Histofine ^®^ (Nichirei Biosciences Cat# 424144, RRID:AB_2868561) for one hour and washed again with PBS. After that, DAB solution (Novolink™, Leica Cat# RE7230-K) was added, and the samples were incubated for ten minutes. DAB solution was discarded and then washed with aquadest. Furthermore, the mayer solution was added into the well and then incubated for 10 minutes and then discarded and washed with aquadest. In a light microscope, expressing IL-6, STAT3 and vimentin proteins will give a brown color, while those that do not express IL-6, STAT3 and vimentin proteins will give a purple/blue color. Immunohistochemistry staining was valued by counting the express protein (brown color) at 20 times field of view at one hepar tissue then the data is averaged and processed statistically. Immunohistochemistry staining was valued by counting the express protein (brown color) at 20 times field of view at every sample then the data is averaged and processed statistically.

### Statistical analysis

Data are expressed as the mean ± standard deviation of each experiment. The values were analyzed by one-way analysis of variance followed by Tukey’s multiple comparison test using GraphPad Prism software version 9 for Windows (GraphPad Prism Software, RRID:SCR_002798, La Jolla, California, USA). Statistical differences were considered significant at the 0.05 levels of probability ( *p*<0.05).

## Result

### *In vivo* result

**Figure 1.**
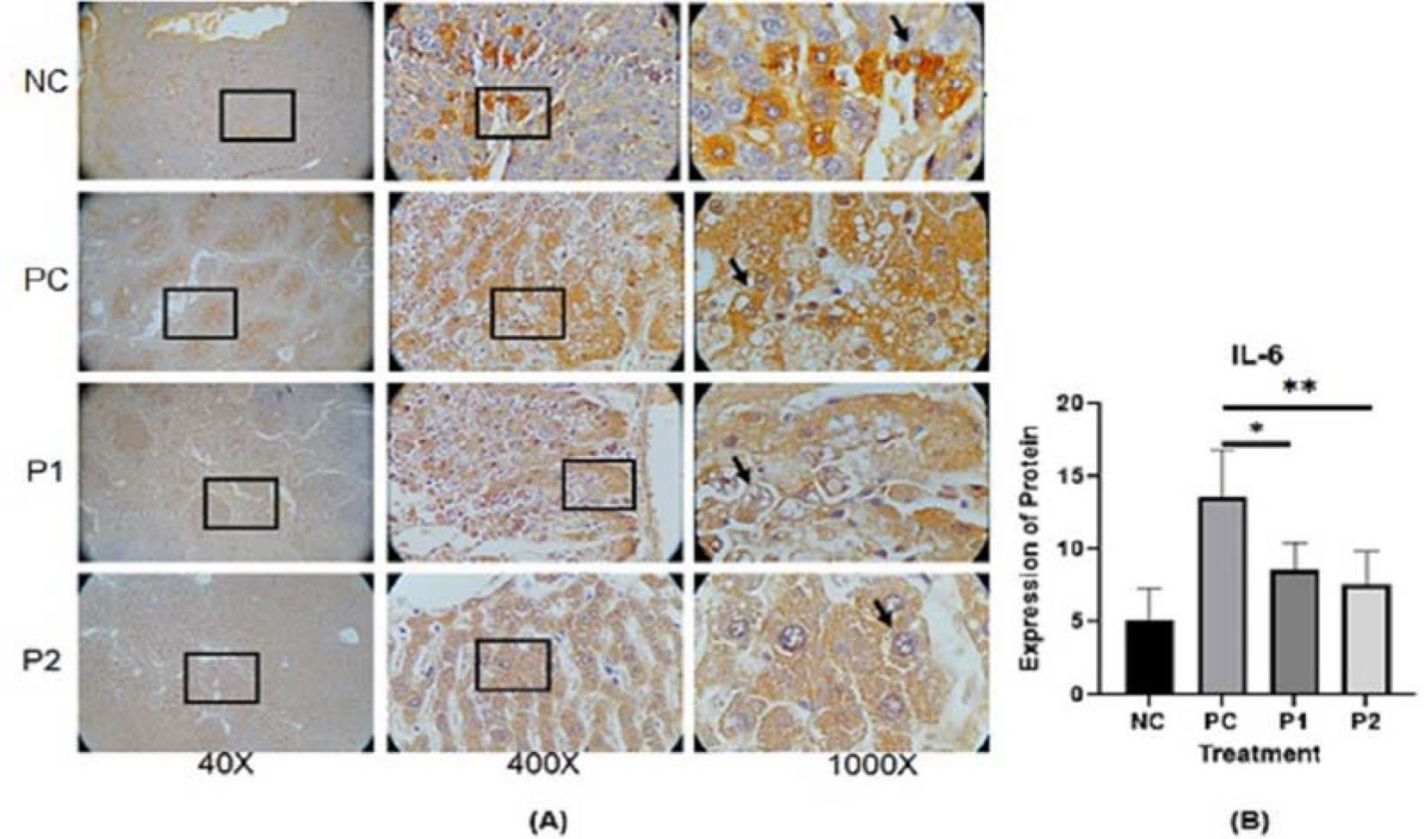
(A) Expression of IL-6 was measured by immunohistochemistry staining in ethanolic extract leaves of *Azadirachta indica* Juss. variant of Indonesia treatment group (P1) and Philippines treatment group (P2) in comparison to the negative control group (NC) and positive control group (PC) at 40×, 400× and 1000× magnification. (B) Expression of IL-6 was analyzed by one-way analysis of variance followed by Tukey’s post hoc test. *p* value<0.05 considered significant.

**Figure 2.**
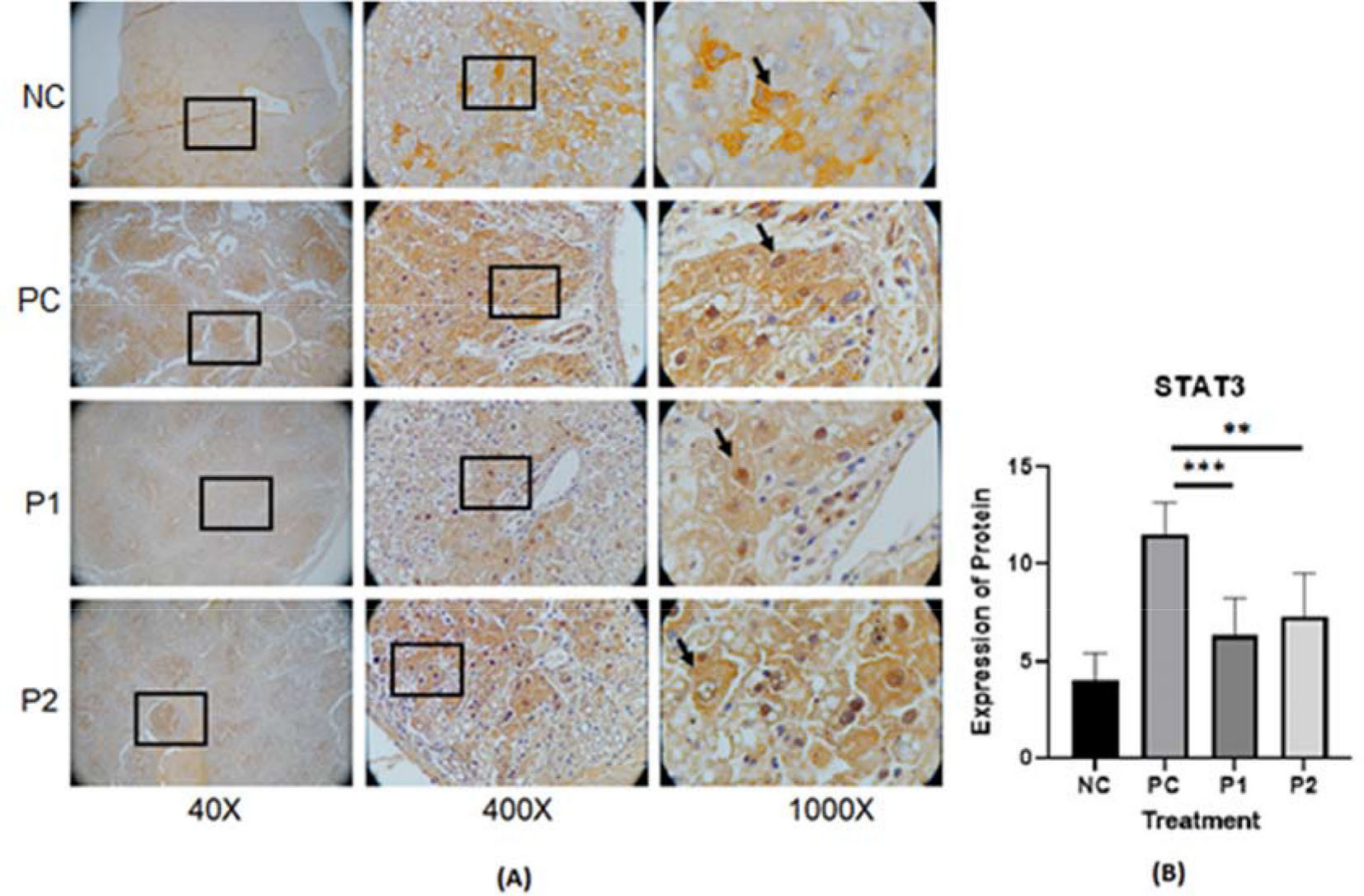
(A) Expression of STAT3 was measured by immunohistochemistry staining in ethanolic extract leaves of *Azadirachta indica* Juss. variant of Indonesia treatment group (P1) and Philippines treatment group (P2) in comparison to the negative control group (NC) and positive control group (PC) at 40×, 400× and 1000× magnification. (B) Expression of STAT3 was analyzed by one-way analysis of variance followed by Tukey’s post hoc test. *p* value<0.05 considered significant.

**Figure 3.**
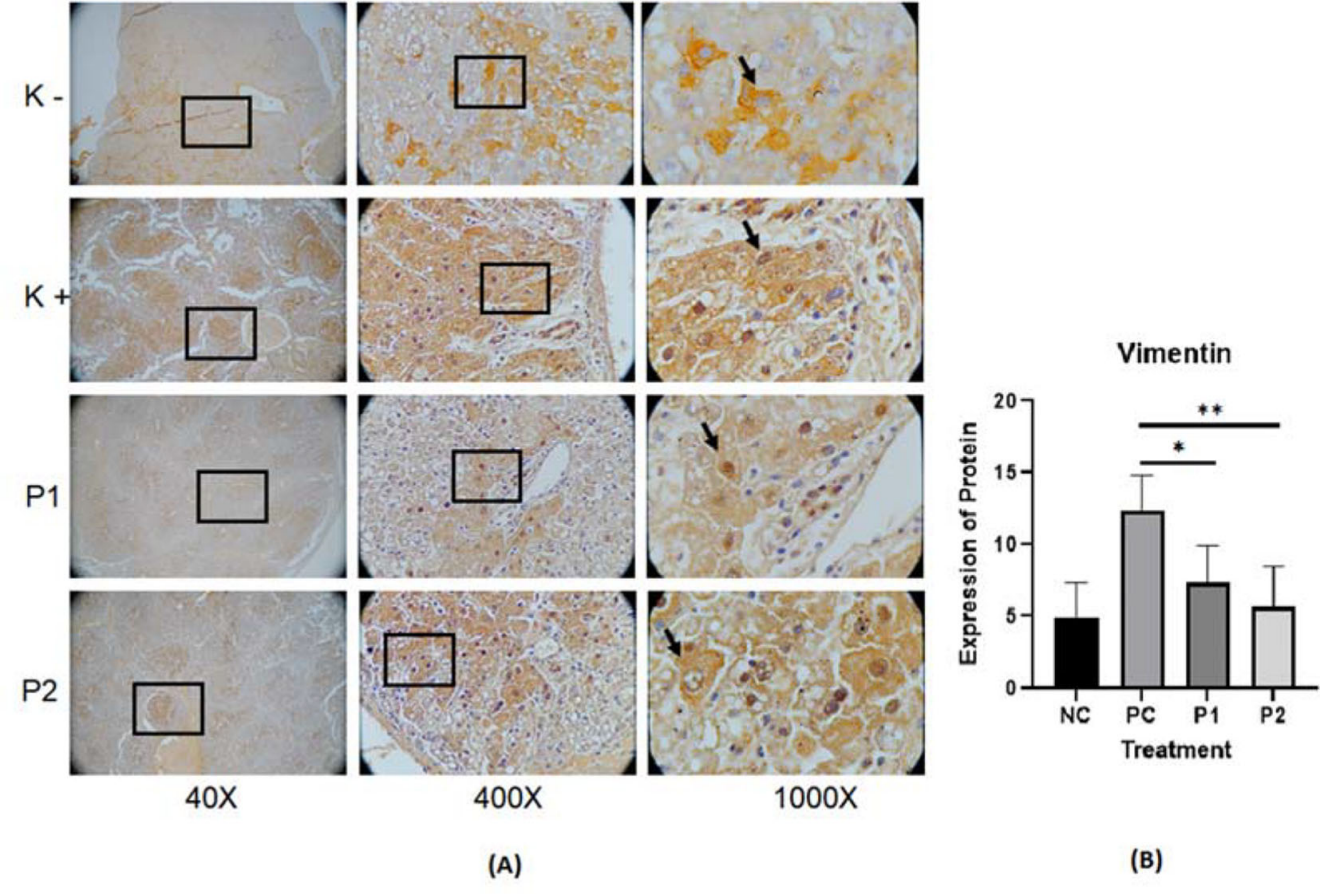
(A) Expression of vimentin was measured by immunohistochemistry staining in ethanolic extract leaves of *Azadirachta indica* Juss. variant of Indonesia treatment group (P1) and Philippines treatment group (P2) in comparison to the negative control group (NC) and positive control group (PC) at 40×, 400× and 1000× magnification. (B) Expression of vimentin was analyzed by one-way analysis of variance followed by Tukey’s post hoc test. *p* value<0.05 considered significant.

The representative image of immunohistochemistry staining for IL-6, STAT3 and vimentin in rat hepar tissue ( Table 1) is shown in Figures 1, 2, and 3. ^37^^–^^39^ Each image depicts a different expression based on the group and the biomarker (IL-6, STAT3 and vimentin).

**Table 1.** Interleukin (IL-6), signal transducer activator of transcription 3 (STAT3) and vimentin by immunohistochemistry staining (mean score ± SD (standard deviation)).

Group	N	IL-6 (Mean ± SD)	STAT3 (Mean ± SD)	Vimentin (Mean ± SD)
Negative control	6	5 ± 2.08	4 ± 1.29	4.8 ± 2.26
Positive control	6	13.5 ± 2.98	11.5 ± 1.50	12.3 ± 2.28
*Azadirachta indica* Juss. variant Indonesia	6	8.5 ± 1.70	6.3 ± 1.69	7.3 ± 2.35
*Azadirachta indica* Juss. variant Philippine	6	7.5 ± 2.14	7.3 ± 1.97	5.6 ± 2.56

### *In vitro* result

**Figure 4.**
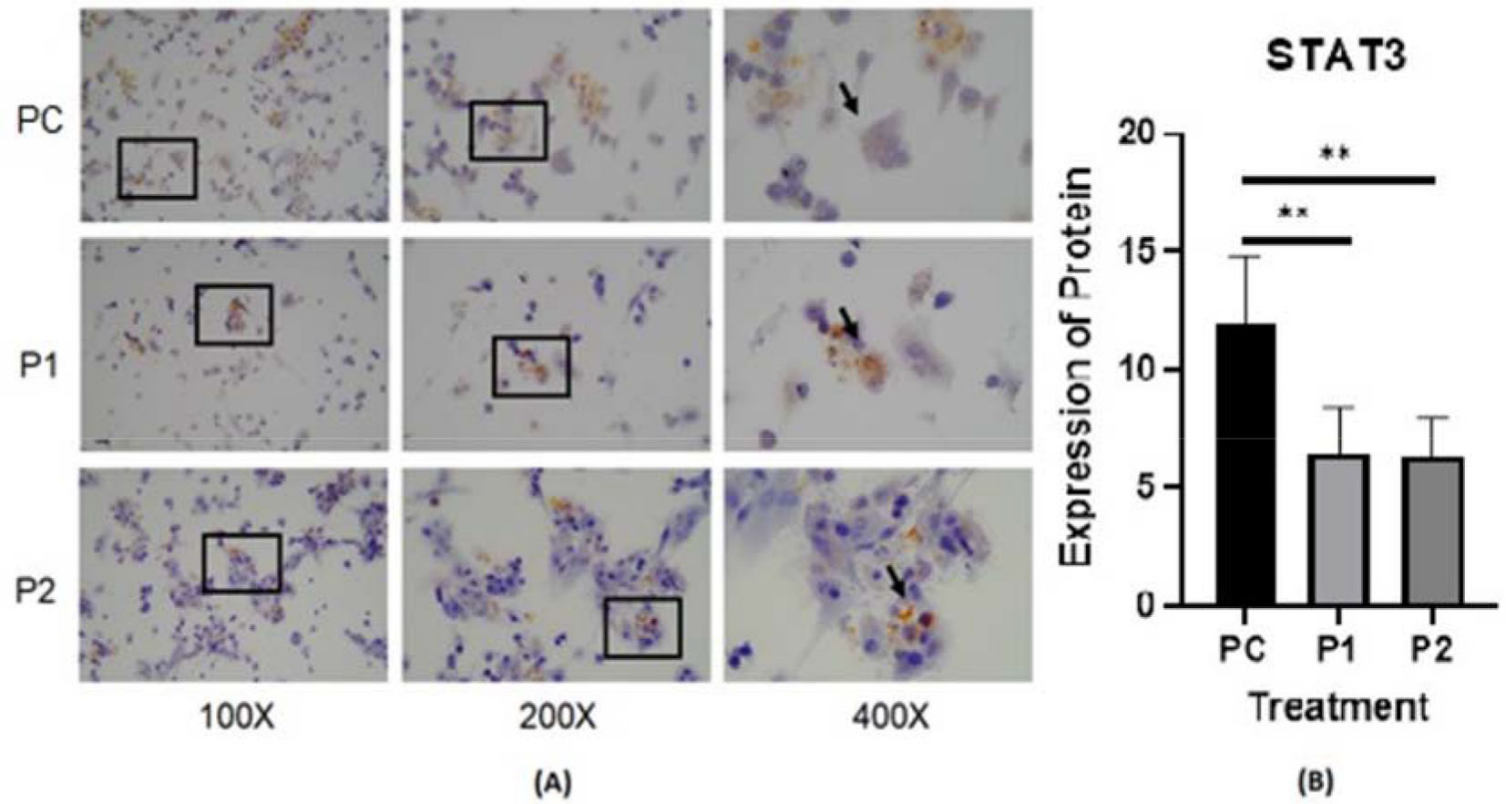
(A) Expression of STAT3 in HepG2 cell line was measured by Immunocytochemistry staining in ethanolic extract leaves of *Azadirachta indica* Juss. variant of Indonesia treatment group (P1) and Philippines treatment group (P2) in comparison positive control group (PC) at 100×, 200× and 400× magnification. (B) Expression of STAT3 was analyzed by one-way analysis of variance followed by Tukey’s post hoc test. *p* value<0.05 considered significant.

**Figure 5.**
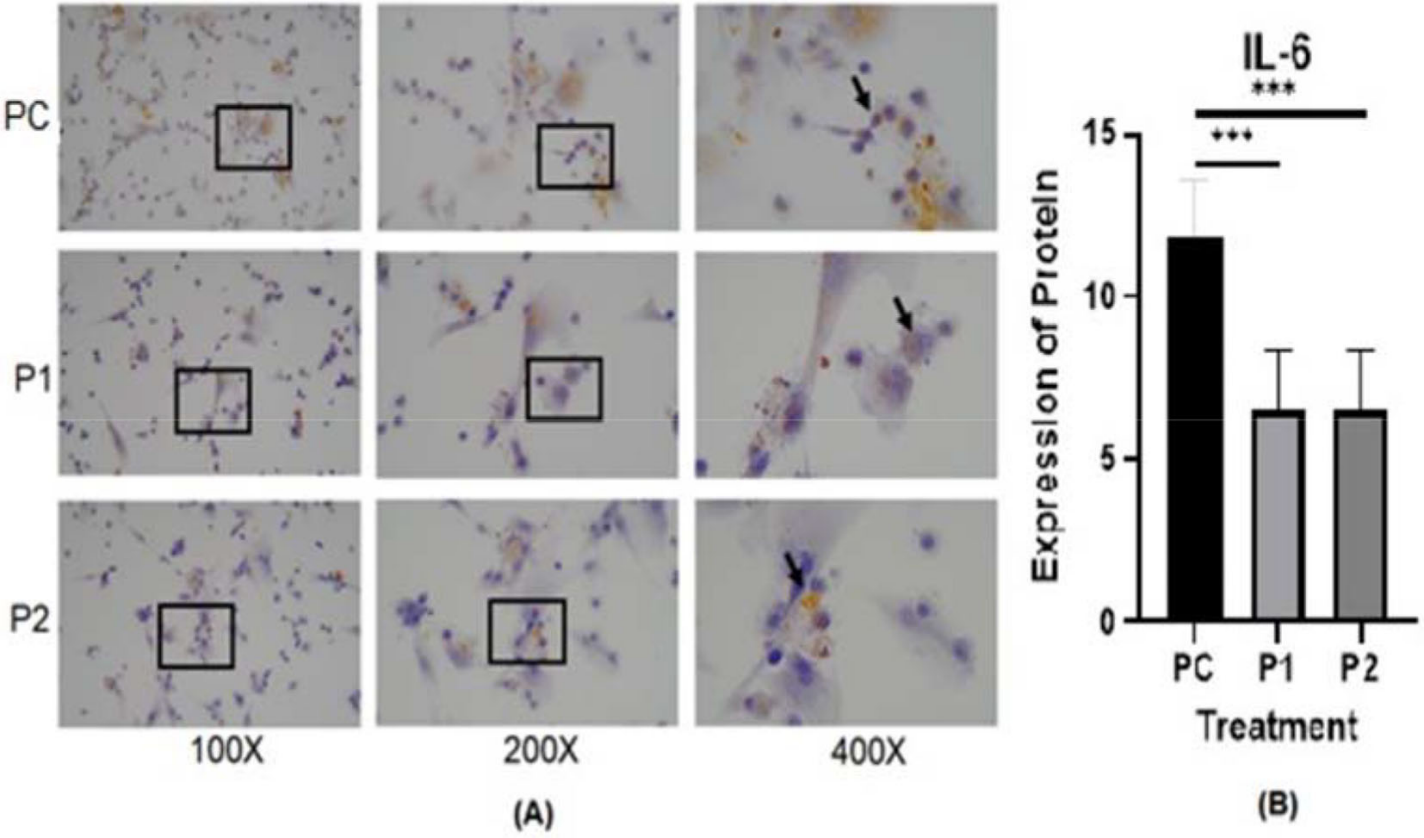
(A) Expression of IL-6 in HepG2 cell line was measured by Immunocytochemistry staining in ethanolic extract leaves of *Azadirachta indica* Juss. variant of Indonesia treatment group (P1) and Philippines treatment group (P2) in comparison positive control group (PC) at 100×, 200× and 400× magnification. (B) Expression of IL-6 was analyzed by one-way analysis of variance followed by Tukey’s post hoc test. *p* value<0.05 considered significant.

**Figure 6.**
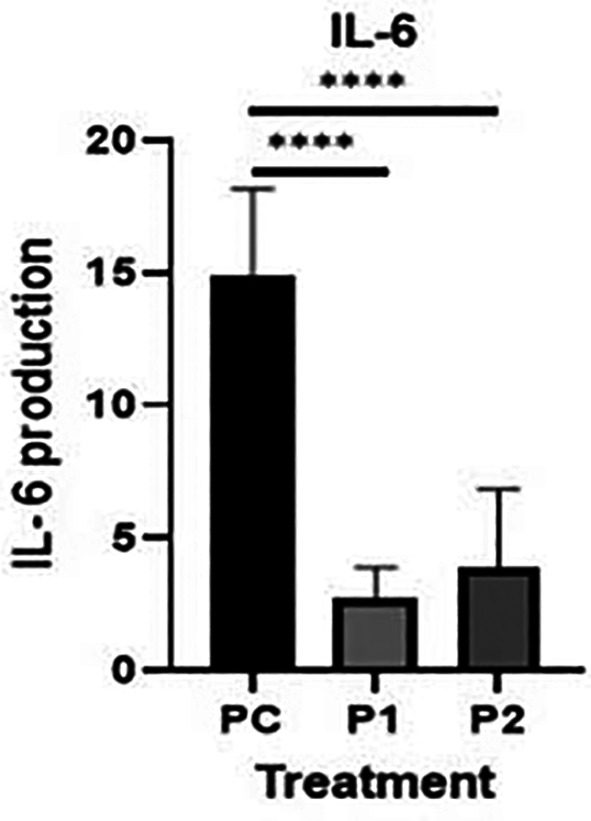
Level of IL-6 proteins in HepG2 cell line was measured by ELISA in ethanolic extract leaves of *Azadirachta indica* Juss. variant of Indonesia treatment group (P1) and Philippines treatment group (P2) in comparison positive control group (PC). The level of IL-6 was analyzed by one-way analysis of variance followed by Tukey’s post hoc test. *p* value<0.05 considered significant.

The representative image of immunocytochemistry staining for IL-6 and STAT3 in HepG2 is shown in Figures 4 and 5. ^40^^,^^41^ The brown color intensity indicates that the related biomarker is expressed at a higher level. Additionally, the level of IL-6 was quantified by the ELISA method ( Figure 6).

Cytokines may contribute to cancer progression. ^42^ In the present study, the IL-6, and STAT3 concentrations in HepG2 cell culture after treatment with Indonesia extract at 170.415 μg/mL and Philippines extract at 170.415 μg/mL were determined ( Table 2). The highest level of inhibition on the release of cytokines was observed in HepG2 for IL-6 compared to untreated positive control cells ( Figure 6). In contrast, there was a slight increase in Indonesia extract group (P1) the release of IL-6 in HepG2 cells than Philippines extract group (P2). In other words, the plant extract caused a significant change in the IL-6 levels and STAT3 expression in HepG2 hepar cancer cells.

**Table 2.** Interleukin (IL-6) and signal transducer activator of transcription 3 (STAT3) by immunocytochemistry staining (mean score ± SD (standard deviation)).

Group	N	IL-6 (Mean ± SD)	STAT3 (Mean ± SD)
Positive control	6	11.83 ± 1.57	12.0 ± 2.58
*Azadirachta indica* Juss. variant Indonesia	6	6.5 ± 1.70	6.5 ± 1.70
*Azadirachta indica* Juss. variant Philippine	6	6.5 ± 1.70	6.3 ± 1.49

## Discussion

On the basis of ethno pharmacological utilization of *Azadirachta indica* in cancer treatment, *Azadirachta indica* variant Indonesia and Philippines was evaluated for its anticancer effect by inhibiting IL-6/STAT-3 signaling pathways. *In vitro* and *in vivo* approaches were used for the explication of possible underlying mechanisms to rationalize the Ayurveda ethno medical uses of the plant from different geographical locations.

*Azadirachta indica* Juss. extract exhibited anticancer effects against DEN and CCl4-induced HCC and hepar cancer cell line (HepG2 cells), while its possible underlying mechanism was estimated through isolated tissue preparations and cells also associated with the reduction in cancer survival. DEN and CCl _4_ are used agents to induce two-step carcinogen protocol in cancer studies. ^36^ Nitrosamine compounds are used as food additives to preserve meat products. ^43^ Nitrosamines can form a genotoxic compound after it is metabolized in the human body by cytochrome P450 in liver. Metabolizing nitrosamine compounds can induce cancer in experimental animals. ^44^^–^^46^ This experiment used the HCC rat model with DEN and CCl _4_-induction, *Azadirachta indica* Juss. leaf extract demonstrated potential of reducing cancer cell survival protective effect against DEN and CCl _4_-induced HCC and hepar cancer cell line (HepG2 cells). *Azadirachta indica* extracts contain an apoptotic constituent which mediates anticancer effect by inhibiting HSC cell growth and inhibiting the IL-6/STAT3 pathway. This study suggests that *Azadirachta Indica* extracts could be a promising technique for inhibiting cancer cell survival.

In this *in vivo* study by immunohistochemistry staining, every treatment group showed a lower expression of IL-6, STAT3 and vimentin in comparison with the positive control groups ( *p*<0.05) ( Table 2). The *in vitro* study by immunocytochemistry staining of expressed IL-6 and STAT3 in HepG2, in every treatment group showed a lower expression of IL-6 and STAT3 in comparison with the positive control groups ( *p*<0.05) ( Table 3). The *Azadirachta indica* Juss. variant Indonesia and Philippines groups exhibited effects on antitumor protein expression by inhibiting IL-6/STAT3 signaling pathway. The *Azadirachta indica* Juss. treatment group was able to significantly decrease IL-6 and STAT3 expression by immunohistochemistry and immunocytochemistry staining with both positive control groups. STAT3 has been shown to be involved in the development of human tumor malignancies. ^36^ STAT3 inhibiting apoptosis or inducing cell proliferation, angiogenesis, invasion and metastasis result in promoting cancer initiation and progression. STAT3 is activated by IL-6-induced dimerization of the IL-6 receptor, which leads to cancer progression in an inflammatory environment. ^47^ Dysregulation of the IL-6-mediated JAK/STAT3 signaling pathway is link to development of several human solid tumors. ^13^^,^^22^^,^^44^^,^^48^

**Table 3.** IL-6 levels by ELISA in HepG2 cell line for Treatment Groups and Control Groups (mean score ± SD (standard deviation)).

Group	N	IL-6 (Mean ± SD)
Positive control	6	14.84 ± 3.02
*Azadirachta indica* Juss. variant Indonesia	6	2.76 ± 1.03
*Azadirachta indica* Juss. variant Philippine	6	3.91 ± 2.69

IL-6 has been implicated as an autocrine stimulator of cancer growth. ^6^^,^^12^^,^^45^^,^^46^ The development of effective therapeutic options requires an understanding of IL-6 survival signaling in malignancies. ^49^^,^^50^ Apoptosis inhibition is a well-studied strategy for cell survival. ^51^ The mechanisms involved in proliferation and acute phase protein synthesis have been thoroughly defined in most investigations of IL-6 signaling in hepatic. ^52^ A study by Bregmann ^12^ showed that one of the essential factors of the development of hepatocellular carcinoma (HCC) is IL-6 trans-signaling. In our study additional data of IL-6 level by ELISA, in every treatment group showed a lower expression IL-6 level in comparison with the positive control groups ( *p*<0.05) ( Table 3). Ethanolic extract leaves of *Azadirachta indica* Juss. variant of Indonesia and Philippines showed a significant decrease in HepG2 and IL-6 levels, when using ELISA method, compared to the positive control group ( Figure 6). It may be said that *Azadirachta indica* leaf extract has a strong effect on reducing the inflammatory cytokine that plays a role in HCC.

Relating to the decreased expression of vimentin protein by immunohistochemistry staining in hepar tissue of *Azadirachta indica* treatment group, it can be said that *Azadirachta indica* treatment decreases the HSCs by knowing the expression of vimentin. The microenvironment of HCC tumors is dominated by HSCs. ^48^^,^^53^^–^^55^ The chronic inflammation is also related to HSC activation. ^50^ HSCs are liver-specific pericytes that play an important role in fibrosis. ^48^^,^^56^^,^^57^ During liver fibrogenesis, HSCs produce enormous amounts of extracellular matrix proteins. ^58^^,^^59^ The most essential function of HSC is in the fibrosis (fibrogenesis) process. Besides that, HSCs also contribute to hepatic inflammation with their ability to secrete and respond to growth stimuli of HCC. ^60^^,^^61^ HSC activation is marked by vimentin protein activation. ^18^^,^^62^

A study by Hu *et al*. ^63^ used immunohistochemistry to investigate the relationship between vimentin overexpression and HCC metastasis utilizing a tissue microarray over 200 primary HCCs and 60 pairs of primary and matched metastatic HCC samples. *Azadirachta indica* Juss. variant Indonesia and Philippines treatment groups had lower expression of IL-6, STAT3 and vimentin that imply a decrease in cell cancer survival.

Therefore, it can be assumed that the *Azadirachta indica* group showed apoptotic effect. The level production IL-6 in HepG2 by ELISA in every treatment group did not show significant difference with the negative control group. Inhibiting HSC cell growth and inhibiting the IL-6/STAT3 pathway could be a promising technique for inhibiting cancer cell survival.

The local growth environment has a strong influence on the quality of medicinal plants. Environmental elements such as precipitation, illumination, temperature, humidity, and soil would fluctuate depending on production location, which can lead to variation in the contents of active ingredients in this medicinal plant. ^64^ This *Azadirachta indica* Juss. leaves variant Indonesia and Philippines were harvested in the same period of time, in November until December, and planted in the same tropical climate. A study by Raissa ^31^ indicated *Azadirachta indica* Juss. variant Indonesia has more flavonoid content than the Philippines one, the Philippines variant has more terpenoids content than the Indonesia one. Despite this difference, the end result showed there is no significant difference of inhibiting STAT3/IL-6 signaling between ethanolic extract leaves of *Azadirachta indica* Juss. variant of Indonesia and Philippines.

There is evidence that bioactive neem chemicals including nimbolide, azadirachtin, and gedunin influence a wide range of biological processes *in vitro* and *in vivo.* ^56^^–^^59^ Both variant have the same effect on inhibiting IL-6/STAT3 signaling, it may be caused by the same amount of main bioactive rather than the variation of bioactive groups such as flavonoid or terpenoids.

This study has limitations, such as administering one dose of the *Azadirachta indica* Juss. variant Indonesia and the Philippines, therefore, the effects could not be compared in a dose-dependent manner. As a result, research with a more variative dose is recommended. Furthermore, more study on other molecular pathways and biomarkers is required to explore the apoptosis potential of both variants in hepatocellular carcinoma.

## Conclusion

A preventive dose of *Azadirachta indica* Juss variant Indonesia and Philippines was able to reduce IL-6/STAT3 expression and decrease vimentin expression in hepar tissue and in HepG2. Our findings show that *Azadirachta indica* Juss variant Indonesia and Philippines, both have cancer-preventive effects in a well-characterized animal model of hepatocellular carcinoma and hepar cancer cell line. Further study including more molecular pathways and biomarkers, is required to explore the mechanism of action of this potential of anti-HCC carcinogenesis by ethanolic extract leaves of *Azadirachta indica* Juss. variant of Indonesia Philippines.

## Data availability

### Underlying data

Figshare: Underlying data for ‘Expression of IL-6 in Rattus norvegicus hepar tissue by Immunohistochemistry staining in treatment group of ethanolic extract leaves of *Azadirachta indica* Juss. variant of Indonesia (P1), Philippines (P2), control group (NC), and positive control group (PC)’, https://doi.org/10.6084/m9.figshare.19367081 ^37^

This project contains the following underlying data:

IL-6 IHC NC 40x.tif

IL-6 IHC NC 400x.tif

IL-6 IHC NC 1000x.tif

IL-6 IHC PC 40x.tif

IL-6 IHC PC 400x.tif

IL-6 IHC PC 1000x.tif

IL-6 IHC P1 40x.tif

IL-6 IHC P1 400x.tif

IL-6 IHC P1 1000x.tif

IL-6 IHC P2 40x.tif

IL-6 IHC P2 400x.tif

IL-6 IHC P2 1000x.tif

Figshare: ‘Expression of STAT3 in *Rattus norvegicus* hepar tissue by Immunohistochemistry staining in treatment group of ethanolic extract leaves of *Azadirachta indica* Juss. variant of Indonesia (P1), Philippines (P2), control group (NC), and positive control group (PC), https://doi.org/10.6084/m9.figshare.19366901. ^38^

This project contains the following underlying data:

STAT3 IHC NC 40x.tif

STAT3 IHC NC 400x.tif

STAT3IHC NC 1000x.tif

STAT3 IHC PC 40x.tif

STAT3 IHC PC 400x.tif

STAT3 IHC PC 1000x.tif

STAT3 IHC P1 40x.tif

STAT3 IHC P1 400x.tif

STAT3 IHC P1 1000x.tif

STAT3 IHC P2 40x.tif

STAT3 IHC P2 400x.tif

STAT3 IHC P2 1000x.tif

Figshare: Raissa, Ricadonna (2022): ‘Expression vimentin in *Rattus norvegicus* hepar tissue by Immunohistochemistry staining in treatment group of ethanolic extract leaves of *Azadirachta indica* Juss. variant of Indonesia (P1), Philippines (P2), control group (NC), and positive control group (PC)’, https://doi.org/10.6084/m9.figshare.19367069. ^39^

This project contains the following underlying data:

Vimentin IHC NC 40x.tif

Vimentin IHC NC 400x.tif

Vimentin IHC NC 1000x.tif

Vimentin IHC PC 40x.tif

Vimentin IHC PC 400x.tif

Vimentin IHC PC 1000x.tif

Vimentin IHC P1 40x.tif

Vimentin IHC P1 400x.tif

Vimentin IHC P1 1000x.tif

Vimentin IHC P2 40x.tif

Vimentin IHC P2 400x.tif

Vimentin IHC P2 1000x.tif

Figshare: ‘Expression of IL-6 in HepG2 cell line by Immunocytochemistry staining in treatment group of ethanolic extract leaves of Azadirachta indica Juss. variant of Indonesia (P1), Philippines (P2) and positive control group (PC)’, https://doi.org/10.6084/m9.figshare.19367081. ^40^

IL-6 ICC NC 40x.tif

IL-6 ICC NC 400x.tif

IL-6 ICC NC 1000x.tif

IL-6 ICC PC 40x.tif

IL-6 ICC PC 400x.tif

IL-6 ICC PC 1000x.tifs

IL-6 ICC P1 40x.tif

IL-6 ICC P1 400x.tif

IL-6 ICC P1 1000x.tif

IL-6 ICC P2 40x.tif

IL-6 ICC P2 400x.tif

IL-6 ICC P2 1000x.tif

Figshare: ‘Expression of STAT3 in HepG2 cell line by Immunocytochemistry staining in treatment group of ethanolic extract leaves of Azadirachta indica Juss. variant of Indonesia (P1), Philippines (P2), and positive control group (PC)’, https://doi.org/10.6084/m9.figshare.19366460. ^41^ ^(p3)^

STAT3 ICC NC 40x.tif

STAT3 ICC NC 400x.tif

STAT3 ICC NC 1000x.tif

STAT3 ICC PC 40x.tif

STAT3 ICC PC 400x.tif

STAT3 ICC PC 1000x.tif

STAT3 ICC P1 40x.tif

STAT3 ICC P1 400x.tif

STAT3 ICC P1 1000x.tif

STAT3 ICC P2 40x.tif

STAT3 ICC P2 400x.tif

STAT3 ICC P2 1000x.tif

### Reporting guidelines

Repository name: ARRIVE checklist for ‘ *In vitro* and *in vivo* study: Ethanolic extract leaves of *Azadirachta indica* Juss. variant of Indonesia and Philippines suppresses tumor growth of hepatocellular carcinoma by inhibiting STAT3-IL-6 signaling’, DOI: https://doi.org/10.6084/m9.figshare.19352474.v2. ^65^

Data are available under the terms of the Creative Commons Attribution 4.0 International license (CC-BY 4.0).

## Author details

Ricadonna Raissa

Roles: Conceptualization, Data Curation, Formal Analysis, Funding Acquisition, Investigation, Methodology, Project Administration, Resources, Software, Supervision, Validation, Visualization, Writing – Original Draft Preparation, Writing – Review & Editing

Wibi Riawan

Roles: Conceptualization, Data Curation, Formal Analysis, Investigation, Methodology, Resources, Software, Supervision, Validation, Visualization, Writing – Original Draft Preparation, Writing – Review & Editing

Anna Safitri

Roles: Conceptualization, Investigation, Methodology, Software, Supervision, Validation, Visualization, Writing – Original Draft Preparation, Writing – Review & Editing

Masruri Masruri

Roles: Conceptualization, Investigation, Methodology, Supervision, Validation, Visualization, Writing – Original Draft Preparation, Writing – Review & Editing

Ma Asuncion Guiang Beltran

Roles: Conceptualization, Formal Analysis, Investigation, Methodology, Supervision, Validation, Visualization, Writing – Original Draft Preparation, Writing – Review & Editing

Aulanni’am Aulanni’am

Roles: Conceptualization, Data Curation, Formal Analysis, Funding Acquisition, Investigation, Methodology, Project Administration, Resources, Software, Supervision, Validation, Visualization, Writing – Original Draft Preparation, Writing – Review & Editing
