# Supplementary material for: In vitro and in vivo study: Ethanolic extract leaves of Azadirachta indica Juss. variant of Indonesia and Philippines suppresses tumor growth of hepatocellular carcinoma by inhibiting IL-6/STAT3 signaling
Source: F1000Res. 2022 Apr 29;11:477. [Version 1] doi: 10.12688/f1000research.109557.1 (PMC10565427; doi:10.12688/f1000research.109557.1)
Supplement: Peer review history

# Reviewer report: Reviewer response for version 1

**DOI:** 10.5256/f1000research.121070.r198761

**Published:** 2023-10-10

**Authors:** Arif Ansori

**Affiliations:** 1 Department of Biology, Universitas Airlangga, Surabaya, East Java, Indonesia; 2 Uttaranchal Institute of Pharmaceutical Sciences, Uttaranchal University, Dehradun, Uttarakhand, India; 3 European Virus Bioinformatics Center, Friedrich Schiller University Jena, Jena, Thuringia, Germany

Firstly, this paper is clear and well-organized. However, I may require some comments on the following issues.

 Title: The title is easy to follow and no mistakes.

 Abstract: This section was well-written and easy to understand. In addition, the objective of the study and the state of the art of the study are clear. It was mentioned by the authors. Furthermore, the keywords should represent the study (such as tumor growth, *Azadirachta indica *Juss., and cancer biology).

 Introduction: Based on my review, I recommend that the authors should add various improvements, such as:

- Hypothesis and objectives (It should be mentioned in the introduction).
- Add a statement or sentence about whether there are any similar studies that have been done before (It should be mentioned in the introduction).
- Add the important issues (It should be mentioned in the introduction).

Material and Methods: This section should be a more detailed protocol or cite the reference. I cannot see it in the methods section. It is important to strengthen your study. Please kindly check all parts of your Methods section.

 Results: This section's part is easy to follow and has no mistakes. I agree about the results.

 Discussion: The discussion section of the study is very comprehensive. But, please kindly add some references to enhance the discussion section (Paragraph 1-3).

 Conclusion: In this part of the manuscript, the authors have successfully established the conclusion of this research. However, it might be helpful if the authors mention the suggestion for forthcoming research. It should make your conclusion more clear.

 References: The core references are acceptable.

Is the work clearly and accurately presented and does it cite the current literature?

Partly

If applicable, is the statistical analysis and its interpretation appropriate?

Yes

Are all the source data underlying the results available to ensure full reproducibility?

Yes

Is the study design appropriate and is the work technically sound?

Yes

Are the conclusions drawn adequately supported by the results?

Yes

Are sufficient details of methods and analysis provided to allow replication by others?

Yes

Reviewer Expertise:

Molecular Biology, Bioinformatics, Cancer Biology, Virology, and Plant Extracts

I confirm that I have read this submission and believe that I have an appropriate level of expertise to confirm that it is of an acceptable scientific standard.

# Reviewer report: Reviewer response for version 1

**DOI:** 10.5256/f1000research.121070.r147588

**Published:** 2022-09-02

**Authors:** Fauzi Yusuf

**Affiliations:** 1 Division of Gastroenterohepatology, Department of Internal Medicine, School of Medicine, Universitas Syiah Kuala, Banda Aceh, Indonesia

I think that this *in vitro* and *in vivo* study is well done. In the *in vitro* study with liver cancer cell line and *in vivo* study with male rats ( *Rattus novergicus*) strain Wistar. The topics Hepatocelluler carcinoma pathways are also interesting and need further study including biomarker and molecular pathways to explore the mechanism of action of many cytokines in supressing immune activation regulator. I think this research article about plant-derived product ( *Azadiracta indica Juss*) will benefit the further study to evaluate the anti cancer properties of ethanolic extract leaves by inhibiting IL-6/STAT3 signalling and another immune activation regulator.

 It seems that the title of this manuscript too strong as the study did not measure the growth of the tumor, because determination of hepatocelluler carcinoma diagnosis in mice is only based on an increase in elevated alpha fetoprotein (AFP).

Is the work clearly and accurately presented and does it cite the current literature?

Yes

If applicable, is the statistical analysis and its interpretation appropriate?

Yes

Are all the source data underlying the results available to ensure full reproducibility?

Yes

Is the study design appropriate and is the work technically sound?

Yes

Are the conclusions drawn adequately supported by the results?

Yes

Are sufficient details of methods and analysis provided to allow replication by others?

Yes

Reviewer Expertise:

Gastroenterology, Hepatology, Microbiota, Short Chain Fatty Acid, Helicobacter pylori, Covid 19, Colorectal cancer.

I confirm that I have read this submission and believe that I have an appropriate level of expertise to confirm that it is of an acceptable scientific standard.
